# Fatigue Fracture Strength of Implant-Supported Full Contour Zirconia and Metal Ceramic Fixed Partial Dentures

**Published:** 2017-05

**Authors:** Fariborz Vafaee, Farnaz Firouz, Masoumeh Khoshhal, Amirarsalan Hooshyarfard, Armaghan Shahbazi, Ghodratollah Roshanaei

**Affiliations:** 1 Associate Professor, Implant Research Center, Department of Prosthodontics, School of Dental Medicine, Hamedan University of Medical Sciences, Hamedan, Iran; 2 Assistant Professor, Department of Prosthodontics, School of Dental Medicine, Hamedan University of Medical Sciences, Hamedan, Iran; 3 Assistant Professor, Implant Research Center, Department of Periodontics, School of Dental Medicine, Hamedan University of Medical Sciences, Hamedan, Iran; 4 Postgraduate Student, Department of Periodontics, School of Dental Medicine, Hamedan University of Medical Sciences, Hamedan, Iran; 5 Postgraduate Student, Department of Prosthodontics, School of Dental Medicine, Hamedan University of Medical Sciences, Hamedan, Iran; 6 Associate Professor, Modeling of Noncommunicable Diseases Research Center, Department of Biostatistics and Epidemiology, School of Public Health, Hamedan University of Medical Sciences, Hamedan, Iran

**Keywords:** Fatigue, Zirconium Oxide, Co-Cr Alloy, Fracture Strength

## Abstract

**Objectives:**

Zirconia restorations have been suggested as a more durable and more appealing alternative to metal restorations. However, their mechanical properties may be negatively affected by fatigue due to superficial stresses or low temperature degradation. This study aimed to assess the fatigue fracture strength of three-unit implant-supported full contour zirconia and pre-sintered cobalt-chromium (Co-Cr) alloy posterior fixed partial dentures (FPDs).

**Materials and Methods:**

In this in-vitro experimental study, 28 posterior three-unit implant-supported FPDs were fabricated of full contour zirconia and pre-sintered Co-Cr alloy, and were cemented on implant abutments. To simulate the oral environment, FPDs were subjected to 10,000 thermal cycles between 5–55°C for 30 seconds, and were then transferred to a chewing simulator (100,000 cycles, 50 N, 0.5 Hz). Afterwards, fatigue fracture strength was measured using a universal testing machine. Data were analyzed by Mann-Whitney U test.

**Results:**

The mean and standard deviation of fracture strength were 2108.6±440.1 N in full contour zirconia, and 3499.9±1106.5 N in pre-sintered Co-Cr alloy. According to Mann-Whitney U test, the difference in this respect was statistically significant between the two groups (P=0.007).

**Conclusions:**

Since the fracture strength values obtained in the two groups were significantly higher than the maximum mean masticatory load in the oral environment, both materials can be used for fabrication of posterior three-unit FPDs, depending on the esthetic demands of patients.

## INTRODUCTION

Porcelain-fused-to-metal (PFM) fixed partial dentures (FPDs) have long been used due to their optimal long-term mechanical properties [1,2], and are the most commonly used type of FPD [3,4]. Despite their favorable properties, they have drawbacks such as corrosion, gingival discoloration adjacent to the crown margin, and unappealing appearance [5]. They cannot truly mimic the translucency and transparency of natural teeth either [6]. Conventional fabrication of a dental prosthetic framework includes several time-consuming steps with high risk of errors from waxing of anatomic contour and cut back to investment and casting [7]. A previous study showed that about 50% of errors in fabrication of implant-supported restorations were due to inaccurate impressions and erroneous casting [8]. Introduction of computer-aided design/computer-aided manufacturing (CAD/CAM) systems revolutionized laboratory fabrication of dental restorations and significantly improved their quality [9–11]. In early phases of introduction of CAD/CAM systems, high-efficiency ceramics and polymers were extensively used, while the use of conventional cobalt-chromium (Co-Cr) alloys was limited due to hardness of alloy blocks, which required costly equipment for milling [12].

In the past, cobalt-chromium-molybdenum (Co-Cr-Mo) alloy could be fabricated by the CAD/CAM process in industrial centers. Two different approaches have been described for restoration fabrication by the CAD/CAM systems using these alloys, namely the additive manufacturing using laser sintering, and the subtractive manufacturing using costly milling machines for milling of high strength materials.

Limited CAD/CAM systems have been designed for dental laboratories, which can cut back the materials in the fully sintered form; however, they are complicated to work with. The newly introduced pre-sintered Co-Cr-Mo alloy available in the market with the brand name Sintron can be used in a desktop milling machine, with faster preparation time and lower cost.

The soft Co-Cr-Mo alloy is first subjected to dry preparation. It contains adhesive materials such as organic coupling agents, which are burned out following sintering at high temperature under argon atmosphere. This results in about 10% reduction in its volume [13]. The CAD/CAM system suggested for the fabrication of metal restorations does not have the shortcomings of other subtractive processes, and can be routinely used in dental laboratories [14].

All-ceramic FPDs are esthetically favorable, and zirconia restorations have been suggested as a durable alternative to metal restorations. However, these restorations must meet the biomechanical requirements, and must have a durability similar to that of PFM restorations [15]. Monolithic yttrium oxide partially stabilized zirconia (Y-TZP) restorations provide acceptable esthetics due to having a superficial glaze layer. High popularity of these crowns for prosthodontic rehabilitation is partly due to their cost-effectiveness. Fabrication of these crowns is easier due to fewer procedural steps, and requires less workforce because they are almost entirely fabricated by the CAD/CAM system [16–18].

Previous studies have reported more advantages for monolithic crowns, including requiring less tooth preparation [17–19], and higher resistance compared to other ceramic materials [16,18,20]. Considering the increased use of zirconia as a monolithic restorative material, and evidence showing that its mechanical properties can be negatively affected by phase transformation due to superficial stresses or low temperature degradation, this study aimed to assess the effect of thermal and mechanical fatigue on fracture strength of full contour zirconia and pre-sintered Co-Cr alloy, since the latter seems to be a suitable alternative to base-metal restorations fabricated by the conventional casting method.

## MATERIALS AND METHODS

In this in-vitro experimental study, sample size was calculated to be 14 restorations in each group according to a previous study [21], assuming type one error of 5% and power of 80%. Thus, 28 three-unit implant-supported FPDs for replacement of the mandibular first molars were fabricated in two groups of full contour zirconia and pre-sintered Co-Cr alloy.

### Preparation of matrix for restorations:

**Fig. 1:**
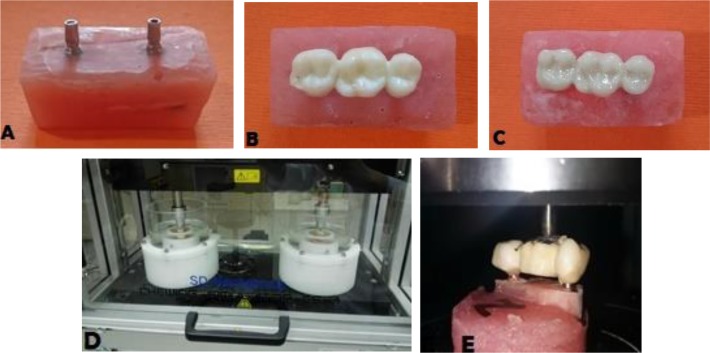
Study process: (A) Placement of implants in resin block. (B) Three-unit fixed partial denture of monolithic zirconia (Zolid). (C) Three-unit fixed partial denture of pre-sintered cobalt-chromium alloy (Sintron). (D) Samples in the chewing simulator. (E) Load application to the samples until failure

Two implants (SICace®, Schilli Implantology Circle, Switzerland) with 4mm diameter and 11.5 mm height were used. The abutment had 4.5 mm diameter and 8.5 mm height with 1.5 mm gingival height. First, the first fixture was mounted in a polymethyl methacrylate resin block (Orthodontic Resin, Dentsply Caulk, USA) using a surveyor. Then, the second fixture was mounted parallel to the first fixture using a surveyor, in a way that the distance between the centers of the two fixtures was 19 mm [22], in order to simulate the missing of mandibular first molar in the clinical setting [21,22]. Titanium abutments were then placed on the implants using 20 N/cm torque (Fig. 1-A). A resin jig (Pattern Resin, GC, Japan) was then fabricated over the abutments. The rest of the samples were mounted using this jig. All 28 resin blocks were randomly divided into two groups of 14 for the fabrication of full contour zirconia and pre-sintered Co-Cr alloy FPDs.

### Fabrication of full contour zirconia FPDs:

The CAD/CAM system was used for the fabrication of these bridges. The fabricated resin block was first scanned to determine the implant site and implant position using a 3D scanner (D700, 3Shape, Denmark), and this scan was processed using 3D Designer software. A bridge with 4×4 mm2 contour dimensions and 12 mm pontic width was fabricated. The minimum thickness was 2 mm. Occlusal anatomy was determined according to the Library 13 of 3Shape software (3Shape Dental system, Copenhagen). Arrangement was done in iCAM version 4.6 software, and the file was transferred to Remote Dental 2.0 software. Next, the full contour zirconium oxide blocks (Ceramill Zoild, Amann Girrbach, Germany) were milled by a milling machine (imes-icore 250i, Germany), and zirconia bridges were sintered in a furnace (Ceramill Therm, Amann Girrbach, Germany) at 1500°C for four hours, according to the manufacturer’s instructions.

### Fabrication of pre-sintered Co-Cr alloy FPDs:

The frameworks were fabricated by the CAD/CAM system as explained earlier for the zirconia group using pre-sintered Co-Cr blocks (Ceramill Sintron, Amann Girrbach, Germany) with the same design as that of the full contour zirconia framework by 1.2 mm cut back for application of porcelain veneering. After completion of preparation of pre-sintered Co-Cr blocks by the milling machine, the frameworks were sintered in a furnace (Ceramill Argotherm, Amann Girrbach, Germany) at 1285°C for six hours under argon atmosphere, according to the manufacturer’s instructions. To ensure that these frameworks have the same design and contour as those of full contour zirconia frameworks, a silicon impression was made of full contour zirconia FPD using putty impression material (C-Silicon, Speedex, Coltene, Switzerland) to provide an index for veneering of the pre-sintered Co-Cr framework. Using this index, opaque and body porcelain (Kuraray Noritake Dental Inc., Japan) were applied on the framework, according to the manufacturer’s instructions.

### Mechanical tests:

Before cementation, the surfaces of FPD and abutments were cleaned with 92% alcohol, and glass ionomer (Gold Label 1, GC, Japan) [23] was used for cementation. One scoop of powder was mixed with two drops of liquid on a mixing pad, according to the manufacturer’s instructions. A plastic spatula was used for mixing the powder and liquid. The entire powder was added to the liquid, and quickly mixed for 20 seconds. Cement was applied to the internal restoration wall, and eventually the FPD was placed on the abutments (Fig. 1-B, C).

After cementation, all the FPDs were subjected to 10,000 thermal cycles (TC/300, Vafaei Industrial Factory, Iran) between 5–55°C (±2°C) for 30 seconds to simulate the oral environment. For dynamic cyclic loading, the FPDs were placed in a chewing simulator (CS-4, Mechatronik, Germany), and were subjected to 100,000 cycles with 50N load and 0.5Hz frequency (Fig. 1-D) [24]. The load was applied to the center of the pontic.

This setting corresponded to two to four months of clinical service of the restoration in the oral cavity [25,26]. Next, the FPDs were transferred to a universal testing machine (Z050, Zwick/Roell, Germany), and were subjected to load application until fracture (Fig. 1-E). Vertical load was applied by a ball-shaped pointer to the center of the pontic at a crosshead speed of 0.5 mm/min [24].

### Data analysis:

Data were analyzed using SPSS version 24 (SPSS Inc., IL, USA). Normality of data was checked using Kolmogorov-Smirnov test. Since data were not normally distributed, non-parametric Mann-Whitney U test was used for statistical analysis. Level of significance was set at 5%.

## RESULTS

Table 1 shows the fatigue fracture strength of full contour zirconia and pre-sintered Co-Cr alloy FPDs.

**Table 1.** Fatigue fracture strength (N) of full contour zirconia and pre-sintered Co-Cr alloy FPDs (n=14)

**Group**	**Mean**	**Median**	**Standard Deviation**	**Minimum**	**Maximum**
**Full Contour Zirconia**	2108.6	2115.6	440.1	1327.7	2869.6
**Pre-sintered Co-Cr alloy**	3499.9	3529.3	1106.5	1762.7	5999.4

Since data were not normally distributed, Mann-Whitney U test was used for statistical analysis, which showed that the mean fatigue fracture strength of pre-sintered Co-Cr alloy was significantly higher than that of full contour zirconia (P=0.007).

## DISCUSSION

Full contour zirconia crowns are becoming increasingly popular due to adequate flexural strength (+1000 MPa), which is higher than the maximum occlusal loading during normal mastication. They have also shown flexural strength higher than 2000 N [27]. Optimal color of restoration, minimal wear of the opposing teeth, conservative preparation, and long-term clinical success are among the advantages of these restorations [28].

Prefabricated dental ceramic blocks are either semi-sintered or fully sintered zirconia blocks [3]. Preparation and cut back of fully sintered YTZP ceramics may cause cracks, and damage the zirconium oxide microstructure [29]. It also requires costly equipment and is time consuming. Thus, semi-sintered zirconia blocks were used in the current study.

Clinical data suggest that defects of all-ceramic FPDs are commonly seen around the connector. An in-vitro study on all-ceramic Y-TZP FPDs with 4×4 mm2 connector dimensions showed that they had a 100% success rate after two years [30]. Therefore, the same connector dimensions were used in the current study.

Beuer et al [16] demonstrated that full contour zirconia had a higher fracture strength than veneered zirconia. Thus, full contour zirconia was used in the present study.

Formation of a strong bond between the opaque layer and alloy is critical for optimal durability and survival of metal ceramic restorations (MCRs). Several studies have demonstrated that increased thickness of the oxide layer covering the alloy is the main factor that decreases the bond strength of MCRs [31–35]. In the study by Stawarczyk et al [13], the thickest superficial oxide layer belonged to Girobond NB casting alloy, while the thinnest layer was noted in Ceramill Sintron; therefore, conventional cast restorations show lower bond strength values than Ceramill Sintron, which can be explained by the effect of the superficial oxide layer on the veneering process.

The authors revealed that Sintron CAD/CAM alloy was comparable to conventional alloys in terms of bond strength [13].

Our study showed that Sintron alloy had significantly higher fracture strength than Zolid. In 2009, Bonfante et al evaluated the prevalence of fracture and defects in three-unit MCR and Y-TZP implant-supported bridges using fatigue testing, and concluded that MCR has higher fracture strength, and is the gold standard for posterior dental areas [22].

In 2013, Eroğlu and Gurbulak evaluated the fatigue and non-fatigue behavior of 60 restorations in three groups of zirconia ceramics (ZC), galvano-ceramics (GC), and PFM on metal dies in canine to second premolar region of the maxilla, and found that ZC had the highest fracture strength (fatigue fracture strength of 2434.9±154.3 N, and non-fatigue fracture strength of 2333.1±183.0 N). Also, ZC group did not show a significant difference between fatigue and non-fatigue behavior. PFM ranked second in this respect (fatigue fracture strength of 1878.5±176.5 N, and non-fatigue fracture strength of 1687.8±162.2 N), and GC ranked last. Significant differences were noted between fatigue and non-fatigue behavior of the latter two groups [24].

In the present study, the mean fracture strength was 2108.6 N in Zolid, and 3499.9 N in Sintron group. Regarding the fracture strength value obtained in zirconia group, we used monolithic zirconia in our study, while Eroğlu and Gurbulak used veneered zirconia. The values in the two studies were in a close range for zirconia, and the existing difference is due to the fact that the bridge fabricated in our study had a 10mm-pontic width to replace the first molar, and was implant-supported; whereas, they fabricated a bridge with a 5mm-pontic width to replace a first premolar, which was supported by a metal die.

Moreover, the zirconia blocks used in the two studies were also of different types. Eroğlu and Gurbulak reported lower strength values than ours for MCRs, which may be attributed to the type of alloy used, and the process of fabrication, which were different in the two studies.

They used Ni-Cr alloy for the fabrication of PFM restorations, while we used Co-Cr alloy, since Ni-Cr has lower strength, and may cause allergic reactions in the clinical setting. Also, Co-Cr alloy has higher strength and corrosion resistance than Ni-Cr. Furthermore, Eroğlu and Gurbulak used the routine casting process, which is time-consuming, and has the risk of procedural errors. In the current study, Co-Cr alloy was milled by the CAD/CAM machine. This process is simple and fast, and has lower risk of errors since it is fully automated. In 2014, Johansson et al reported that monolithic Y-TZP ceramics showed significantly higher fracture strength than other groups (2795 and 3038 N). The fracture strength of veneered Y-TZP group (2229 N) was significantly higher than that of monolithic lithium disilicate and veneered highly translucent Y-TZP groups [20].

The fracture strength value reported by Johansson et al, for monolithic zirconia group was higher than that in our study, which may be due to the following reasons: First, they evaluated single crowns, while we evaluated three-unit posterior bridges. Second, Johansson et al. performed fatigue test by applying 10,000 cycles at 10° angle with 1Hz frequency, while we performed fatigue test by using 100,000 cycles applied vertically to the pontic with 0.5Hz frequency. Third, the monolithic zirconia blocks used in the two studies were from different commercial brands. Finally, it appears that higher fracture strength in Sintron group is due to higher fracture strength of its Co-Cr framework.

On the other hand, since fractures in Sintron group were in the form of porcelain chipping in our study, it seems that thinner superficial oxide layer and the homogenous and uniform surface of this alloy provide adequately strong bond to porcelain, which leads to higher fracture resistance. In monolithic zirconia group, after thermocycling and cyclic loading, the zirconia surface is exposed to liquids, and undergoes low temperature degradation.

Consequently, the energy required for transformation toughening (responsible for final strength of zirconia) decreases. Moreover, during cyclic loading, zirconia undergoes macro and micro-cracks due to its brittle nature, which gradually jeopardize its strength, and result in fracture under lower magnitudes of load.

## CONCLUSION

In the present study, the fracture strength values found for Zolid and Sintron were higher than the reported maximum mean masticatory load in the oral environment. Thus, both types of restorations can be successfully used for patients requiring three-unit posterior bridges, depending on their esthetic requirements.
